# Retesting for severe acute respiratory coronavirus virus 2 (SARS-CoV-2): Patterns of testing from a large US healthcare system

**DOI:** 10.1017/ice.2020.413

**Published:** 2020-08-10

**Authors:** Amy J. Kennedy, Mary K. Hilmes, Linda Waddell, Alexandrea B. Bartow, Carla M. Baxter, Christiane M. Hadi, Graham M. Snyder, Jessica S. Merlin

**Affiliations:** 1 Division of General Internal Medicine, University of Pittsburgh School of Medicine, Pittsburgh, Pennsylvania; 2 Wolff Center at the University of Pittsburgh Medical Center, Pittsburgh, Pennsylvania; 3 Department of Cardiothoracic Surgery, University of Pittsburgh Medical Center, Pittsburgh, Pennsylvania; 4 Division of Infectious Diseases, University of Pittsburgh School of Medicine, Pittsburgh, Pennsylvania

*To the Editor—*Coronavirus disease 2019 (COVID-19), a respiratory illness caused by severe acute respiratory syndrome coronavirus 2 (SARS-CoV-2), has caused a global pandemic, leading to significant morbidity and mortality.^1,2^ Accurate testing is essential to the identification and treatment of new cases of COVID-19 in the inpatient and outpatient settings.

In the United States, the initial focus of COVID-19 testing has been on ensuring adequate access to large-scale testing via a public health approach. However, given the limitations in efforts to ensure widespread access, individual hospitals and healthcare systems have worked to ensure that enough tests are available to meet clinical demand. Often decisions on who to test are left to individual clinicians, which leads to questions about when and who to retest for COVID-19, how often false positives or negatives might occur, and the duration of positivity.^3^

Research regarding why retesting for SARS-COV-2 might be indicated or what results might be expected is lacking. This report describes patterns of SARS-CoV-2 nucleic acid polymerase chain reaction (PCR) retesting in inpatients and outpatients within a large US healthcare system. We aimed to learn more about potential reasons for retesting and test characteristics.

## Methods

We performed a retrospective chart review of all inpatients and outpatients aged ≥18 years receiving care within the University of Pittsburgh Medical Center (UPMC) with ≥2 SARS-CoV-2 PCR tests with an initial test between March 3 and May 3, 2020, and a subsequent test before May 21, 2020. UPMC operates 40 academic, community, and specialty hospitals and 700 doctors’ offices and outpatient sites across Pennsylvania, New York, and Maryland. Widespread testing within UPMC at individual clinician discretion became available in March 2020, and recommended asymptomatic screening of preoperative patients began in May 2020.

We collected demographic characteristics, setting of care, reason for retesting, certain COVID-19 risk factors (ie, nursing home resident, immunocompromised, healthcare worker, COVID-19 exposure, travel history), and the date of tests, allowing for calculation of time between tests. PCR testing was performed using a lab-derived assay and through a commercial laboratory.

Descriptive statistics were performed overall and for 4 groups: (1) initial positive test, any subsequent result(s) positive; (2) initial positive test, any subsequent result(s) negative; (3) initial negative test, any subsequent result(s) negative; and (4) initial negative test, any subsequent result(s) positive. These groups were not mutually exclusive and were constructed to learn as much as possible about testing characteristics. For example, within group 1, the potential length of time a test *could* remain positive (even if a subsequent test was then negative). The University of Pittsburgh Institutional Review Board approved this study.

## Results

Among >30,000 initial tests, 485 were repeated; 259 were inpatients (53.6%) and 230 were outpatients (46.7%) at the time of initial test. Most individuals (348, 71.9%) had 2 tests and 136 (28%) had ≥3 tests. Most patients were white (78%), aged 41–80 years (71.6%), and had symptoms of fever (35.1%), cough (37.2%), or shortness of breath (32.0%) at baseline (Table 1).

**Table 1.** Sample Characteristics of 485 Participants With Repeat SARS-CoV-2 Testing

Characteristic	Total (N = 492)^a^	Group 1: Initial Positive, Any Subsequent Positive (N = 35), No. (%)	Group 2: Initial Positive, Any Subsequent Negative (N = 39), No. (%)	Group 3: Initial Negative, Any Subsequent Negative (N = 403), No. (%)	Group 4: Initial Negative, Any Subsequent Positive (N = 15), No. (%)
Sex, male	208 (42.3)	21 (60.0)	22 (56.4)	157 (39.0)	8 (53.3)
**Age, median y**	60.5	63	58	56	65
0–20	6 (1.2)	1 (2.9)	1 (2.6)	4 (1.0)	0 (0.0)
21–40	100 (20.3)	6 (17.1)	8 (20.5)	83 (20.6)	3 (20.0)
41–60	176 (35.8)	7 (20.0)	11 (28.2)	155 (38.5)	3 (20.0)
61–80	176 (35.8)	18 (51.4)	18 (46.2)	133 (33.0)	7 (46.7)
≥81	34 (6.9)	3 (8.6)	1 (2.6)	28 (6.9)	2 (13.3)
**Location at time of initial test**					
Outpatient	259 (52.6)	22 (62.9)	20 (51.3)	210 (52.1)	7 (46.7)
Inpatient	230 (46.7)	13 (37.1)	19 (48.7)	190 (47.1)	8 (53.3)
**COVID-19 risk factor**					
COVID-19 exposure	73 (14.8)	9 (25.7)	12 (30.8)	45 (11.2)	7 (46.7)
Healthcare worker	63 (12.8)	3 (8.6)	2 (5.1)	56 (13.9)	2 (13.3)
Nursing home resident	44 (8.9)	5 (14.3)	8 (20.5)	28 (6.9)	3 (20.0)
Travel history	14 (2.8)	6 (17.1)	4 (10.3)	4 (1.0)	0 (0.0)
Immunocompromised	36 (7.3)	2 (5.7)	4 (10.3)	30 (7.4)	0 (0.0)
Days between tests, median (IQR)	13 (2–18)	18 (10.5–23.5)	23 (17–29)	4 (3–15)	8 (2–14)
**Reason for retesting**					
Clinical suspicion	108 (22.0)	2 (5.7)	6 (15.4)	96 (23.8)	4 (26.7)
Asymptomatic screen	154 (31.3)	0 (0.0)	0 (0.0)	153 (38.0)	1 (6.7)
Disposition request	44 (8.9)	5 (14.3)	8 (20.5)	28 (6.9)	3 (20.0)
Discontinue isolation	20 (4.1)	4 (11.4)	10 (25.6)	6 (1.5)	0 (0.0)
Test entered in error	8 (1.6)	0 (0.0)	0 (0.0)	8 (2.0)	0 (0.0)

a Some patients fell into >1 result group, hence the total of 492 > 485; 492 = number of entries analyzed; 485 = number of unique entries.

Among 74 patients with an initial positive test, 35 (47%) had any subsequent positive result (group 1) and 39 (53%) had any subsequent negative result (group 2). The median time between an initial and last positive test was 18 days (interquartile range [IQR], 13; range, 2–39), and the median time between an initial positive and first negative test was 23 days (IQR, 12; range, 3–43). The most common reason for repeat testing was inpatient discharge planning, followed by discontinuation of inpatient isolation (Table 1).

Among 418 patients with an initial negative test, only 15 (3.6%) had any subsequent positive result (group 4), while 403 (96.4%) had any subsequent negative result (group 3). The most common reason for repeat testing was preoperative asymptomatic screening (N = 154, 31.3%), followed by clinical suspicion for a false negative (N = 108, 22.0%). For those who went from negative to positive, median time between tests was 8 days (IQR, 12; range, 1–23).

## Discussion

In this retrospective study of a large US healthcare system, we found that retesting for SARS-CoV-2 was uncommon and often resulted in multiple negative tests. Most individuals were retested due to preprocedural asymptomatic screening or clinical suspicion for COVID-19 disease. In this population, PCR positivity persisted for a median of 18 to 23 days, and repeat testing after an initial negative test infrequently yielded a positive result. Prior studies have suggested that PCR positivity may persist beyond symptoms or infectivity; our findings suggest a potential time frame for this persistence.^4^ Most repeat tests ordered after an initial negative test were also negative, which is consistent with other emerging findings.^5,6^

The main limitation of this study is that testing was conducted only in individuals in whom it was clinically indicated, and only at the clinician’s discretion, which limited our ability to draw conclusions about differences between test groups or to calculate a true false-negative rate.

In summary, we found that retesting for SARS-CoV-2 was rare and usually resulted in multiple negative tests. Future research should work to identify predictors of initial false negatives and to provide a more refined estimation of duration of infectivity.
